# Fidelity of DNA polymerases in the detection of intraindividual variation of mitochondrial DNA

**DOI:** 10.1080/23802359.2019.1697188

**Published:** 2019-12-12

**Authors:** Paulo Cseri Ricardo, Elaine Françoso, Maria Cristina Arias

**Affiliations:** Departamento de Genética e Biologia Evolutiva – Instituto de Biociências, Universidade de São Paulo, São Paulo, Brazil

**Keywords:** *Taq* DNA polymerase, Q5 DNA polymerase, heteroplasmy, NUMTs

## Abstract

Here we investigated the consequences of PCR amplification errors in the identification of intraindividual mtDNA variation. The bumblebee *Bombus morio* was chosen as model for the *COI* gene amplification tests with two DNA polymerases (*Taq* and Q5) presenting different error rates. The amplifications using *Taq* resulted in a significant increase of singleton haplotypes per individual in comparison to Q5. The sequence characteristics indicated that *Taq* resulted haplotypes are mostly due to amplification errors. Studies focusing on intraindividual variability should address special attention to the DNA polymerase fidelity to avoid overestimation of heteroplasmic haplotypes.

## Introduction

*Taq* DNA polymerase (*Taq*-pol) is the most common polymerase used in polymerase chain reaction (PCR) (Yamagami et al. 2014). This enzyme was isolated first from the thermophilic bacterium *Thermus aquaticus* (*Taq*) (Saiki et al. 1988) and is thermostable. This characteristic was decisive for the automation of PCR (Reiss et al. 1990), besides the increase in specificity and efficiency of the reaction (Saiki et al. 1988). Over time, other thermostable DNA polymerases have been isolated from other microorganisms (e.g. *Pfu* and *Vent*^®^) or developed from protein engineering (e.g. Phusion^®^ and Q5^®^). Some of these enzymes have 3′-5′ proofreading exonuclease activity, absent in *Taq*-pol, which promotes the checking and removing mismatched nucleotides during the polymerization (Kunkel 1992; Kunkel and Bebenek 2000) and, therefore, are considered as high-fidelity DNA polymerases.

The fidelity of a DNA polymerase is usually expressed by the mean error rate per base per duplication (Keohavong and Thilly 1989). *Taq*-pol has an error rate between 2 × 10^−4^ (Saiki et al. 1988; Keohavong and Thilly 1989) and 2 × 10^−5^ (Eckert and Kunkel 1990; McInerney et al. 2014). Proofreading activity of the high-fidelity polymerases drops error rates to 10^−6^ or even lower (Cline 1996; Li et al. 2006; McInerney et al. 2014). Despite the higher error rate of *Taq*-pol compared to high-fidelity polymerases, the frequency of each error in the population of amplified molecules is still considered low (Lin et al. 2002). However, these errors may hinder the analysis of coamplification of different sequences (Pascual et al. 1994; Bracho et al. 1998), as in studies of heteroplasmy.

In a previous study, heteroplasmy was unveiled for the bumblebee *Bombus morio* (Hymenoptera, Apidae, Bombini) (Françoso et al. 2016). Thus *B. morio* was our model species to evaluate the degree of DNA polymerases error effects in the identification of heteroplasmic haplotypes. Here, we tested two DNA polymerases (*Taq*-pol and Q5), each presenting distinct error rates, for the amplification of a mitochondrial fragment encompassing the cytochrome C oxidase subunit I (*COI*) gene.

## Material and methods

### Samples and DNA extraction

Six individuals of *B. morio* (1BM, 2BM, 3BM, 4BM, 5BM, and 6BM), from four Brazilian locations, were used for DNA extraction following the protocol described by Françoso et al. (2015). Samples were obtained from the cryogenic collection (−80 °C) of the Laboratório de Genética e Evolução de Abelhas from Instituto de Biociências, Universidade de São Paulo, São Paulo, Brazil (see Supplementary file 1).

### Amplification

A 676 bp fragment of *COI* gene was amplified with a *Taq*-pol and a high-fidelity polymerase for each individual in a Mastercycler pro (Eppendorf, Germany), using the primers BarbeeF (Françoso and Arias 2013) and mtD9 (Simon et al. 1994).

#### Amplifications with Taq DNA polymerase

The amplifications were set up with 4.0 μl of DNA template in 20 μl final volume containing 1X PCR buffer, 0.4 μM each primer, 0.2 mM each dNTP, 1.5 mM MgCl_2_, and 1.5 U of Platinum^®^ *Taq* DNA Polymerase (Thermo Fisher Scientific, Waltham, MA). Reactions consisted of an initial denaturation at 94 °C for 2 min followed by 35 cycles at 94 °C for 45 s, 48 °C for 45 s, and 64 °C for 50 s, and a final extension at 64 °C for 5 min. The error rate of Platinum *Taq* is 2.28 × 10^−5^ according to the manufacturer.

#### Amplifications with high-fidelity DNA polymerase

Amplifications were run in a 25 µl volume containing 12.5 µl of Q5^®^ High-Fidelity 2X Master Mix (New England Biolabs, Ipswich, MA), 0.8 µM of each primer and 4.0 µl of DNA template. PCR reactions were conducted according to Q5 manufacturer’s recommendations, and included an initial denaturation at 98 °C for 30 s followed by 35 cycles at 98 °C for 10 s, 50 °C for 30 s, and 72 °C for 30 s, and a final extension at 72 °C for 2 min. The error rate of Q5 polymerase is 5.3 × 10^−7^ (Potapov and Ong 2017).

### Cloning and sequencing

PCR products from each polymerase amplification were cloned in the pGEM plasmid vector (Promega, Madison, WI) and used to transform competent *Escherichia coli* DH5-α cells. For each *B. morio* sample and DNA polymerase tested, around 48 colonies were picked, added to a 20 μl of TE buffer (0.5X) and boiled at 99 °C for 10 min. The cloned inserts were amplified using the pUC/M13 primers Forward (17mer) (−40) (5′-GTTTTCCCAGTCACGAC-3′) and Reverse (17mer) (5′-CAGGAAACAGCTATGAC-3′), following the conditions previously described for *Taq*-pol. PCR products were purified with the ExoProStar (GE Healthcare Life Sciences, Little Chalfont, UK) and sequenced at the Macrogen sequencing service (Macrogen Inc., Seoul, South Korea).

### Quantification of intraindividual haplotypes

Sequences were aligned using the algorithm MUSCLE (Edgar 2004) in GENEIOUS 9.1.6 software (Kearse et al. 2012). The number of haplotypes per individual was quantified using PEGAS 0.9 package (Paradis 2010) in the R 3.3.1 (R Core Team 2016). Haplotype networks were constructed in the POPART 1.7 program (Leigh and Bryant 2015), using the median-joining algorithm (Bandelt et al. 1999).

### Frequency of errors and characterization of base substitutions

The frequency of PCR products with an amplification error was estimated using the following equation adapted from Smith and Modrich (1997):

(1) f=lna

where *f* is the expected frequency of molecules exhibiting an error, *l* the size of the amplified fragment (in bp), *n* the number of cycles used in the amplification, and *a* the polymerase error rate. These estimates were used to calculate the number of expected sequences with an amplification error, as follows:

(2) es=fN

were *es* is the number of expected sequences with an amplification error, *f* the expected frequency of molecules exhibiting an error, and *N* the number of sequenced clones. These estimates were compared with the number of intraindividual haplotypes to test whether the observed results can be explained by amplification errors. These comparisons were performed using Student’s *t*-test implemented in the R 3.1.1.

The number of synonymous and non-synonymous substitutions between intraindividual haplotypes was calculated by DNAsp 5.10.01 (Librado and Rozas 2009). Transitions and transversions were also quantified to verify if the amount of substitutions is related to the mutational spectrum of the polymerases.

## Results and discussion

**Figure 1.**
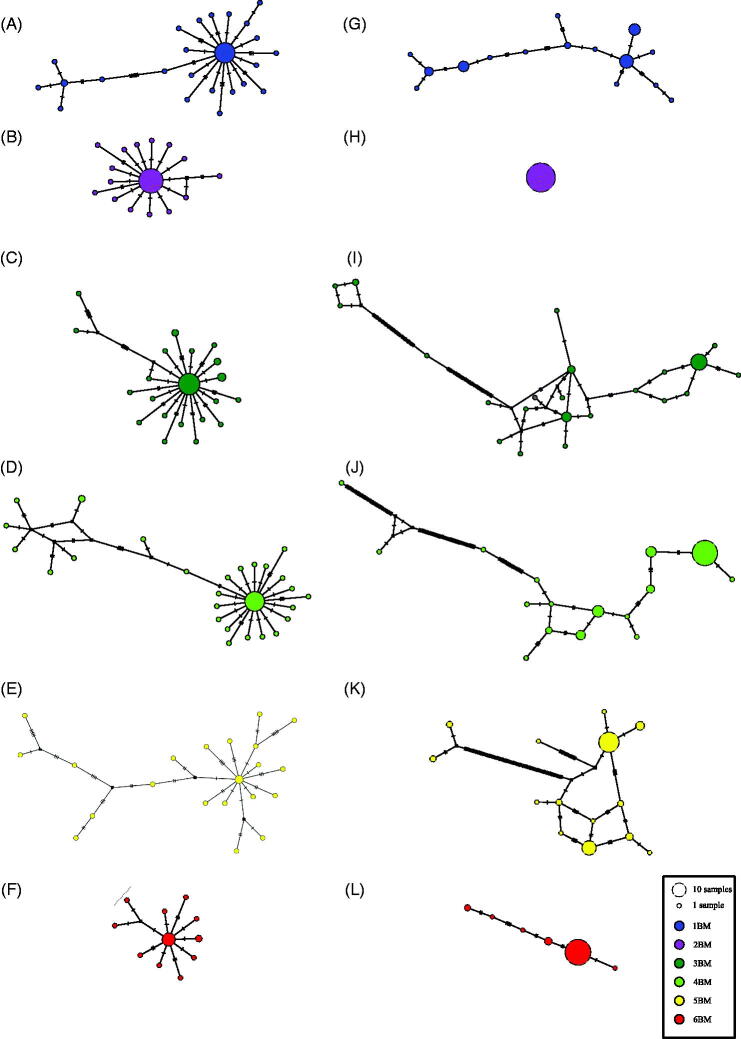
The six intraindividual (see aside legend) haplotype networks obtained from *COI* sequences cloned of *Bombus morio* (GenBank accession: MK994547-MK994748). *COI* amplifications were performed using two different polymerases: Platinum *Taq* DNA Polymerase (A–F) and Q5 DNA polymerase (G–L). Circles represent intraindividual haplotypes, and the size is proportional to their frequency. Crossbars indicate number of nucleotide substitution. Black squares represent a missing intermediate haplotype.

The two polymerases tested recovered several *COI* haplotypes per individual (GenBank accession: MK994547-MK994748). However, the results indicate that most of the singletons (haplotypes represented by a single intraindividual sequence) obtained after *Taq*-pol probably are due to amplification errors. First, 90% of intraindividual haplotypes are singletons (Table 1), and most present only a single base substitution in relation to the most frequent intraindividual haplotype, leading to a star-like topology network (Figure 1(A–F)). Second, no significant difference was verified between the number of intraindividual haplotypes and the expected number of sequences with amplification errors (Table 1). This statistical significance remained when only singletons were considered. And third, most of the observed substitutions among singleton were A→G/T→C transitions (61.4%) (Supplementary file 2). Previous studies have reported that about 57 to 66% of the errors generated by *Taq*-pol are A→G/T→C transitions (Dunning et al. 1988; Ennis et al. 1990; Bracho et al. 1998; Potapov and Ong 2017), values similar to those observed in this work. Besides that, the number of non-synonymous substitutions was also higher among PCR products amplified with *Taq*-pol (mean: 18 *Taq*, 3 Q5). In addition, indels were observed in several sequences (Table 1). Usually the substitutions verified in heteroplasmic sequences are synonymous and occur in the third base of the codon. Amplification errors, in turn, are randomly distributed in the sequence, regardless the codon position (Bracho et al. 1998). Indels may also indicate amplification errors, especially in coding regions due to reading frameshift, leading to nonfunctional gene products, normally.

**Table 1.** Individual identification and respective number of sequenced clones (*N* seq), haplotypes (*h*), singletons (*s*), type of substitutions (synonymous [*syn*] and non-synonymous [*nsyn*]) and indels recovered after DNA amplification using *Taq* and Q5. Singletons frequencies in relation to the total haplotype number (%), and the number of expected sequences considering the amplification error (*es*) are presented.

	*Taq* DNA polymerase							High-fidelity polymerase (Q5)						
Individual	*N* seq	*h*	*s*	*es*	*syn*	*nsyn*	indels	*N* seq	*h*	*s*	*es*	*syn*	*nsyn*	indels
1BM	43	24	22 (92%)	23	19	17		39	15	10 (67%)	<1	12	5	
2BM	42	19	18 (95%)	23	9	10	5	44	1	0 (0%)	<1			
3BM	45	21	17 (81%)	24	17	26	2	42	23	19 (83%)	<1	12^a^	6^a^	1^a^
4BM	45	30	28 (93%)	24	17	24	4	59	17	11 (65%)	<1	8^a^	4^a^	1^a^
5BM	23	21	20 (95%)	12	21	20	2	54	14	6 (43%)	<1	9^a^	2^a^	0^a^
6BM	19	11	9 (82%)	10	5	9	1	43	6	3 (62%)	<1	9	0	
Total/Mean	217/36	126/21	114/19	117/19	88/15	106/18	14/2	281/46	76/12	49/8	4/	50/8	17/3	2/0

^a^Sequences treated as NUMTs were excluded to avoid bias, since they presented high number of base substitutions.

Conversely, the clones sequencing data from high-fidelity polymerase Q5 presented fewer singletons in comparison to *Taq*-pol. The difference between the singletons frequency obtained with *Taq*-pol and with Q5 (90 and 53%, respectively) was significant (*p*-value = 0.00384). Also, the star-like pattern was not observed in the Q5 haplotype networks (Figure 1(G–L)). Strikingly, the individual 2BM showed only a single haplotype (Figure 1(H)). The singletons obtained with the Q5 usually presented more than one substitution relative to the most frequent intraindividual haplotype, but the genetic distances typically did not exceed 2%. Considering the Q5 error rate, the frequency of singletons for most samples was higher than expected (Table 1). Moreover, most of the singletons presented several substitutions in relation to the most frequent haplotypes. Thus, it is unlikely that the singletons are due to amplification errors solely. They may represent real mitochondrial haplotypes. It is noteworthy that some singletons from individual 3BM, 4BM and 5BM presented a high number of base substitutions (Figure 1(I–K)), and the intraindividual genetic distances ranging from 2.6 to 11.8%. In addition, these singletons showed many non-synonymous substitutions relative to the other sequences. It is quite likely that these singletons represent NUMTs, copies of mitochondrial regions present in the nuclear genome (Richly and Leister 2004), since the differences in relation to the other sequences cannot be explained solely by amplification errors.

Finally, the number of singletons presented a narrow and inverse relation to the fidelity of the polymerase, especially the ones linked to the most frequent haplotype by just one nucleotide substitution. Therefore, these singletons probably result from amplification errors and may lead to misinterpretation concerning mtDNA variation (false positives). Singletons with many substitutions probably represent intraindividual haplotypes, since the accumulation of many errors in the single sequence is unlikely, even using low fidelity polymerases. Thus, in order to reduce false positives and the chances of removing real intraindividual haplotypes we suggest (1) the use of a high-fidelity enzyme, which can greatly reduce the number of singletons, and (2) the removal of singletons, but only those linked by a single substitution to the frequent haplotypes.

## Supplementary Material

Supplemental Material
