# Supplementary material for: Fidelity of DNA polymerases in the detection of intraindividual variation of mitochondrial DNA
Source: Mitochondrial DNA B Resour. 2019 Dec 12;5(1):108–12. doi: 10.1080/23802359.2019.1697188 (PMC7720943; doi:10.1080/23802359.2019.1697188)
Supplement: Supplemental Material [file TMDN_A_1697188_SM4436.zip › Supplementary file 1.docx]

Supplementary file 1. *Bombus morio* samples used in this study and their respective voucher ID and collection information.

| **Individual** | **Voucher^a^** | **Collection site** | **Coordinates** | **Collect year** |
| --- | --- | --- | --- | --- |
| 1BM | EF369 | Presidente Nereu | 27°15’34’’S 49°19’46’’W | 2013 |
| 2BM | EF416 | Caxias do Sul | 29°8’3’’S 51°13’4’’W | 2013 |
| 3BM | 290309 | Igrapiúna | 13°40’54’’S 39°9’53’’W | 2008 |
| 4BM | 290309-2314 | Igrapiúna | 13°40’54’’S 39°9’53’’W | 2011 |
| 5BM | EF208 | Igrapiúna | 13°40’54’’S 39°9’53’’W | 2011 |
| 6BM | EF348 | Brasília | 15°45’41’’S 47°52’18’’W | 2012 |

^a^ Specimens kept in the cryogenic collection (-80 °C) of the Laboratório de Genética e Evolução de Abelhas from Instituto de Biociências, Universidade de São Paulo, São Paulo, Brazil.

Supplementary file 2. Distribution of nucleotide substitution types verified among the singletons (number in parenthesis) according to each DNA polymerase used.

|  | **Transitions (%)** | |  | **Transversions (%)** | | | |
| --- | --- | --- | --- | --- | --- | --- | --- |
| **Polymerase** | A→G/T→C | G→A/C→T |  | A→C/T→G | A→T/T→A | G→C/C→G | G→T/C→A |
| *Taq* (114) | 61.4 | 11.7 |  | 4.1 | 18.0 | 0.7 | 4.1 |
| Q5 (49) | 35.3 | 47.0 |  | 5.9 | 0.0 | 5.9 | 5.9 |
